# PSYCHOTIC DISORDER DUE TO PSYCHOSOCIAL STRESS EPISODE. REVIEW OF A CASE.

**DOI:** 10.1192/j.eurpsy.2023.2207

**Published:** 2023-07-19

**Authors:** A. Gonzalez-Mota, I. M. Peso-Navarro, C. Garcia-Cerdan, C. Munaiz-Cosio, M. Ligero-Argudo, C. Martin-Gomez

**Affiliations:** 1 Psychiatry, University Hospital Complex of Salamanca; 2 Institute of Biomedicine of Salamanca (IBSAL); 3 Psychiatry, School of Medicine, University of Salamanca, Salamanca, Spain

## Abstract

**Introduction:**

Psychotic disorder is defined as a loss of contact with reality. Those who suffer from it perceive an altered reality, assuming it to be true.This feeling of unreality generates nervousness, anguish, hypervigilance and even social and emotional isolation.

We present the case of a 18-year-old woman who attended the Emergency Department accompanied by the director of her college due to behavioral alterations. The patient reports that since she has moved to Salamanca to study,she has the feeling that her father has hired spies, one of them being her classmate, being able to hear sounds and voices, which she defines as motivating her to go on with her life. She reports that she is in a lower mood in this context and that there have been some days when she has not been able to attend class.

**Objectives:**

The objectives are to study the severity of the psychotic disorder in a young patient subjected to an episode of stress and to observe the reaction of the patient when it has been properly treated.

**Methods:**

We carry out a review of the clinical history of a 18-year-old female patient with psychotic disorder, admitted to the Psychiatric Brief Hospitalization Unit (PBHU) in Salamanca.

**Results:**

The patient was treated with Risperidone 2mg/24h. After a few days in the PBHU, total disappearance of the psychotic symptoms was observed and the patient is completely self-critical. Once she was discharged, it was decided that she should return home with her parents for several months and continue treatment with Aripiprazole and Sertraline.

**Conclusions:**

Occasionaly, there are ethical dilemmas about beginning to treat young patients with psychotic ideas derived from external situations. Optimal treatment including drugs, psychotherapy and family support are essential. According to the scientific literature,a greater involvement in diagnosis,treatment and follow-up is recommended in patients with psychotic symptomatology derived from stress.

**Disclosure of Interest:**

None Declared

